# Cytoskeleton reorganization mediates alpha6beta1 integrin-associated actions of laminin on proliferation and survival, but not on steroidogenesis of ovine granulosa cells

**DOI:** 10.1186/1477-7827-3-19

**Published:** 2005-05-16

**Authors:** Frédérique Le Bellego, Stéphane Fabre, Claudine Pisselet, Danielle Monniaux

**Affiliations:** 1 Physiologie de la Reproduction et des Comportements, UMR 6175 INRA-CNRS-Université de Tours-Haras Nationaux, INRA 37380 Nouzilly, France

## Abstract

**Background:**

Laminin (LN) is one of the most abundant extracellular matrix components of the basal lamina and granulosa cell layers of ovarian follicles. Culture of ovine granulosa cells (GC) on LN substratum induces cell spreading, enhances cell survival and proliferation, and promotes luteinization. Previous investigations have shown that these effects are mostly mediated by the alpha6beta1 integrin, but its signalization pathways have not been investigated. This study aimed to assess the importance of the cytoskeleton in the alpha6beta1 integrin-mediated actions of laminin on survival, proliferation and steroidogenesis of ovine GC.

**Methods:**

The relationships between morphology and functions of ovine GC cultured on substrata containing LN or/and RGD peptides were investigated. The effects of (1) cytochalasin D, an actin cytoskeleton-disrupting drug, (2) a specific function-blocking antibody raised against alpha6 integrin subunit (anti-alpha6 IgG), and (3) an inhibitor of the ERK1/2 signalization pathway (PD98059) were assessed for GC shape, pyknosis and proliferation rates, oestradiol and progesterone secretions.

**Results:**

Cytoskeleton disruption by cytochalasin D induced cell rounding, inhibited proliferation, promoted pyknosis, inhibited progesterone secretion and enhanced oestradiol secretion by GC cultured on LN. When GC were cultured on various substrata containing LN and/or RGD peptides in the presence or absence of anti-alpha6 IgG, both the existence of close correlations between the percentage of round cells, and the GC proliferation rate (r = -0.87) and pyknotic rate (r = 0.76) were established, but no relationship was found between cell shape and steroidogenesis. Inhibition of the ERK1/2 signalization pathway by PD98059 had no effect on GC shape, proliferation or pyknotic rates. However, it dramatically reduced progesterone secretion, expression of cytochrome P450 cholesterol side-chain cleavage and 3beta-hydroxysteroid deshydrogenase enzymes, and enhanced oestradiol secretion, thereby reproducing all the effects of the anti-alpha6 IgG on steroidogenesis of GC cultured on LN.

**Conclusion:**

LN may participate in the paracrine control of follicular development through different mechanisms. It could enhance proliferation and survival of GC through its alpha6beta1 integrin-mediated actions on cytoskeleton. In contrast, its stimulating action on GC luteinization could be partly mediated by the ERK1/2 pathway, irrespective of cell shape.

## Background

Follicular development is under the control of both gonadotropins and numerous paracrine factors that are critically involved in determining the fate of follicles, atresia or ovulation. From the primordial to the preovulatory follicular stage, the outer layer of granulosa cells (GC) lays on a basal lamina that separates them from the theca layers and interstitial ovarian tissue [1]. This basal lamina, consisting of extracellular matrix (ECM) components such as laminin (LN), fibronectin, collagens and various glycoproteins and proteoglycans, is subjected to intense remodeling during follicular development and atresia, changing its composition from the primordial to the preovulatory or atretic stages [2]. For example, the basal lamina becomes less collagenous and more laminin-rich during follicular development [3,4]. In antral follicles, laminin and other ECM components are also present within the multilayered wall of GC [5,6], particularly in basal lamina-like material deposited as aggregates between the GC layers, recently called focimatrix (for focal intra-epithelial matrix) [7]. These observations indicate that ECM components contribute to the microenvironment of GC, but their specific roles in follicular development have not yet been established.

LN is one of the most abundant ECM components of the basal lamina [2,4,5,8-12] and, as stated above, it is also present within the granulosa layers of antral follicles. In sheep, LN levels increase considerably in the granulosa of antral follicles during the follicular and preovulatory phases of the cycle [6]. *In vitro *experiments have shown that LN improves GC survival (rat: [13]; sheep: [14]) and stimulates the proliferation of GC from small antral follicles [14]. In GC from large antral and preovulatory follicles, LN increases progesterone secretion (rat: [13,15]; pig: [16]; sheep:[6]) and decreases estradiol secretion [6], suggesting that it might promote luteinization. Overall, these results suggest that LN has an important regulatory effect on GC functions throughout the terminal development of antral follicles.

Among the different integrins that can bind LN and mediate its action in various cell types, α6β1 and α6β4 have the particular feature of being highly specific LN receptors [17]. The α6 integrin subunit has been shown to be greatly expressed in GC of different animal species (human: [18]; marmoset: [19,20]; pig: [21]; mouse: [22]; sheep: [6]). In sheep, GC of healthy antral follicles express high levels of α6β1 integrin, and when a function-blocking antibody raised against the α6-integrin subunit is added to the medium of GC cultured on LN, their survival, proliferation and steroidogenesis are dramatically altered [6]. These results suggest that α6β1 integrin mediates most LN actions on GC, but the mechanisms involved in α6β1 integrin-mediated functional changes in GC are unknown.

From previous observations, addition of the antibody raised against the α6-integrin subunit in the GC culture medium impairs cell-spreading on LN substratum and induces the formation of clusters of rounded cells [6]. It can be hypothesized that changes in cell shape might be responsible for all or part of the functional changes observed in survival, proliferation and steroidogenesis of GC. It has been established in various cell models that integrin binding to ECM components promotes changes in the mechanical tension of the cytoskeleton and thereby induces multiple signaling pathways [23,24]. The cytoskeleton consists of actin microfilaments, microtubules and intermediate filaments which connect to form a three-dimensional network that runs from the plasma membrane, and particularly from integrins, to the chromosomes in the nucleus [25]. The importance of the cytoskeleton in mediating steroidogenesis in response to gonadotropins has been suggested [26-33]. It is likely that cell morphology influences cell polarization and organelle organization through the cytoskeleton, thereby controlling steroid production and secretion [28,30,34], but the possible role of integrin-mediated cytoskeleton changes has not yet been established in regulating GC steroidogenesis, survival and proliferation.

This study aimed to assess the importance of the cytoskeleton in the α6β1 integrin-mediated actions of LN on survival, proliferation and steroidogenesis of ovine GC. For this purpose, different experiments were performed to investigate the existence of coupling between cell shape and function when α6β1 integrin activity was altered. Firstly, the action of cytochalasin D, an actin cytoskeleton-disrupting drug, was studied on both the shape and functions of GC cultured on LN substratum. Secondly, the action of a function-blocking antibody raised against α6 integrin subunit was studied on both the shape and functions of GC cultured on substrata containing different ratios of LN and RGD peptides that specifically recognize the α5/ α8/ αv/ αIIb but not the α6 integrin subfamilies [35]. Lastly, the consequences of inhibiting the ERK1/2 (Extracellular signal-Related kinase) signalization pathway, that has been shown to transduce some of the α6β1 integrin-mediated effects of LN [36-39], were studied on both GC shape and functions.

## Methods

### Reagents and chemicals

Fluorogestone acetate sponges used to synchronize estrous cycles were obtained from Intervet Pharma (Angers, France). Porcine FSH (pFSH) from pituitary extract (pFSH activity = 1.15 × activity NIH pFSH-P1) used for animal injections was obtained from Dr. Y. Combarnous (Nouzilly, France). B2 medium for cell cultures was prepared according to Menezo [40]. Rat monoclonal antibody GoH3 raised against human α_6 _integrin subunit (anti-α_6 _IgG) for use in cell cultures was purchased from Serotec (Oxford, England). For western immunoblotting, rabbit polyclonal antibody raised against cytochrome P450 cholesterol side-chain cleavage (anti-P450scc) was purchased from Chemicon (Euromedex, Mundolsheim, France), rabbit polyclonal antibody raised against 3β-hydroxysteroid deshydrogenase (anti-3βHSD) was a gift of Dr. V. Luu-The (Quebec, Canada) and rabbit polyclonal antibody raised against ERK1 and ERK2 (anti-ERK1/2) was purchased from Santa Cruz (Le Perray-en-Yveline, France). Rabbit polyclonal antibody raised against the phosphorylated forms of ERK1 and ERK2 (anti-P-ERK1/2) was purchased from Calbiochem (Meudon, France). Anti-rabbit IgG antibody coupled to horseradish peroxidase was purchased from Interchim (Montluçon, France). The following reagents were purchased from Sigma (L'Isle d'Abeau Chesnes, France): McCoy's 5a medium with bicarbonate, penicillin/streptomycin, bovine serum albumin (BSA tissue culture grade) used for culture medium, transferrin, selenium, bovine insulin, androstenedione, LN from EHS tumor (mainly LN-1: [41]), cytochalasin D, FITC-conjugated phalloidin, PD98059, Igepal, phenylmethylsulfonyl fluoride (PMSF), leupeptin, aprotinin, sodium fluoride, sodium pyrophosphate and sodium orthovanadate. Hepes, L-glutamine, fungizone and trypsin were purchased from GIBCO BRL (Cergy-Pontoise, France). RGD peptides (arginin – glycin – aspartic acid sequence) were obtained from Interchim (Montluçon, France). Sterile 96-well plates (Nunclon Delta) were obtained from Nunc (Naperville, IL, USA) and plastic tissue-culture chamber slidesfrom Poly-Labo (Strasbourg, France). [^3^H]thymidine (specific activity 6.7 Ci/nmol) was obtained from Dupont De Nemours (Les Ulis, France) and NTB2 emulsion for autoradiography from Integra Bioscience (Cergy-Pontoise, France). For protein assay, the BC Assay protein kit was obtained from Uptima Interchim (Montluçon, France). Immobilon P membranes for western blots were obtained from Millipore Corporation (Bedford, MA, USA). ECL (enhanced chemiluminescence) reagents were obtained from Amersham Pharmacia Biotech (Orsay, France).

### Animals

All procedures were approved by the Agricultural and Scientific Research agencies (approval number A 37801) and conducted in accordance with the guidelines for Care and Use of Agricultural Animals in Agricultural Research and Teaching. Experimental research was performed with the approval of the regional ethics committee of the Région Centre (Tours, France). During the reproductive season, adult Romanov ewes were treated with intravaginal sponges impregnated with progestagen (fluorogestone acetate, 40 mg) for 15 days to mimic a luteal phase. GC were collected from animals slaughtered in the luteal phase of the following estrous cycle (10 days after sponge removal), after treatment with intramuscular injections of 6 IU and 5 IU pFSH administered 24 h and 12 h prior to slaughter respectively.

### Isolation of GC

For each culture experiment, immediately after slaughter, ovaries from 3 ewes were immersed for 15 min in isotonic solution containing amphotericin (50 mg/ml) and antibiotics (2 million UI/ml penicillin and 2 g/l streptomycin). The ovaries were placed in B2 medium, and follicles larger than 1 mm in diameter were dissected within 1 hour of slaughter. A total number of 50–70 small (1–3 mm in diameter) and 10–15 large (> 4 mm in diameter) were dissected from these pooled ovaries. Follicular fluid from large follicles (> 4 mm) was aspirated with a 26-gauge needle. Each follicle was then slit open in B2 medium, and GC were removed by gently scraping the interior surface of the follicle with a platinum loop. GC suspensions were pooled by follicle size (small: 1–3 mm, or large: > 4 mm). The two resulting cell suspensions were centrifuged at 300 × g for 7 min and re-suspended in culture medium (McCoy's 5a containing bicarbonate supplemented with 20 mmol/l Hepes, 100 kUI/l penicillin, 0.1 g/l streptomycin, 3 mmol/l L-glutamine, 0.1% BSA (w/v), 100 μg/l insulin, 0.1 μmol/l androstenedione, 5 mg/l transferrin, 20 μg/l selenium). The total number of cells per suspension was estimated by counting an aliquot of each suspension using a hemocytometer under a phase-contrast microscope. The number varied between 10 × 10^6 ^and 20 × 10^6 ^cells per suspension. Cell viability, determined after vital staining with trypan blue dye (0.125%, final concentration) varied between 60 and 80%.

### GC culture

GC culture was performed according to Campbell's method [42]. GC from small and large follicles were cultured in 96-well tissue-culture plates or in tissue-culture chamber slides coated with LN (5 μg/cm^2 ^in distilled water), unless specified. In the experiments using culture substrata containing LN and/or RGD peptides, different mixes were prepared using the LN solution described above and an RGD peptide solution (1.67 μg/cm^2 ^in PBS) to obtain different ratios of LN and RGD peptides (100%, 43%, 25%, 18%, 14%, 0% w/w % of LN in the mix used for coating). The concentrations of the peptides had been determined in preliminary experiments for their morphological effects on GC cultured in the presence or absence of anti-α_6 _IgG (0.5 μg/ml). The different substrata were prepared 72 h before use and allowed to dry at room temperature.

GC suspensions from small and large follicles were seeded at 10^5 ^viable cells/well and cultured at 37°C in a humidified atmosphere with 5% CO_2_, in serum-free culture medium (see isolation of GC). The effect of cytochalasin D, an inhibitor of actin polymerization, was tested at different concentrations in the 0.05 – 5 μg/ml range. The effect of PD98059, an inhibitor of the ERK1/2 activation pathway, was tested in the 1 – 30 μM range. The effects of anti-α_6 _IgG (0.5 μg/ml) were always compared with the effects of the inhibitors within the same culture experiment. Each condition was tested in triplicate in each GC culture from small and large follicles. Culture media were partially replaced (175 μl out of 250 μl) every 48 h and the spent medium was stored at -20°C until assay. At 144 h of culture, cells were detached with trypsin and counted as described above (see isolation of GC), or prepared for western immunoblotting. For studies of ERK1/2 phosporylation, GC were cultured on LN or plastic, with and without inhibitors, for 24 h before western immunoblotting. In preliminary experiments, this culture time was shown to be needed for cell plating on substratum [14] and allowed detection of early effects of LN on ERK1/2 phosporylation.

### Determination of thymidine labeling index and pyknotic index

GC proliferation and survival were assessed by measuring the thymidine labeling index (percentage of labeled cells) and the pyknotic index (percentage of pyknotic cells) after 48 h of culture. This has been shown to allow the be the optimal culture time for the study of the effects of various factors, particularly ECM components, on both proliferation and survival of cultured ovine GC [6,14,43].

To determine the thymidine labeling index, cells were washed with B2 medium without thymine, then incubated with [^3^H]thymidine (0.25 μCi/ml) at 37°C for 2 h. After 2 washes with B2 medium (with thymine), cells were detached with 1% trypsin, pelleted and fixed in 3% glutaraldehyde for 1.5 h at room temperature. Cells were then smeared onto histological slides by cytocentrifugation. Smears were stained with Feulgen, dipped in NTB2 emulsion, air-dried, and exposed for autoradiography for 6 days at 4°C. The thymidine labeling index was estimated by counting the number of labeled and unlabeled cells in 20 different microscopic fields (100X objective).

To determine the pyknotic index, cells were detached by trypsin, fixed in glutaraldehyde, cytocentrifuged, and smears were stained with Feulgen as described above. The pyknotic index was estimated by counting the number of pyknotic cells, i.e. cells with condensed or fragmented nuclear chromatin [44], and non-pyknotic cells in 20 different microscopic fields (100X objective). Previous results have established that the presence of pyknotic cells is associated with DNA fragmentation characteristic of apoptotic process in cultured GC [14].

For both the thymidine labeling index and the pyknotic index, calculations were made on 500–1000 cells per slide.

### Estradiol-17β and progesterone radioimmunoassays

The concentrations of estradiol and progesterone in the culture medium of GC from large follicles were measured after 144 h of culture. This has been shown to be the optimal culture time for the study of the effects of ECM components on steroidogenesis of cultured ovine GC [6,14]. The radioimmunoassay protocol previously described [45-47] was adapted to measure steroids in cell culture media directly. The estradiol detection limit was 1.5 pg/tube (7.5 pg/well) and the intra- and inter-assay coefficients of variation were less than 7% and 9% respectively. The progesterone detection limit was 12 pg/tube (60 pg/well), and the intra- and inter-assay coefficients of variation were less than 10% and 11% respectively. Results were expressed as the amount of steroids secreted between 96 h and 144 h of culture per 50,000 cells recovered at the end of the culture period.

### Western immunoblotting

GC whole extracts were obtained by resuspension in 100 μl cell lysis buffer [150 mM NaCl, 50 mM Tris-HCl (pH 7.5), 1% Igepal, 0.5% sodium deoxycholate, 0.1% SDS] containing several protease inhibitors (10 mM PMSF, 1 μg/ml leupeptin, 1 μg/ml aprotinin) and phosphatase inhibitors (100 mM sodium fluoride, 10 mM sodium pyrophosphate, 2 mM sodium orthovanadate) at 4 °C for 20 min. Cell lysates were centrifuged at 20,000 × g for 20 min, and the protein concentration in the supernatants was determined by the BC Assay protein kit following the manufacturer's protocol. Aliquots (5 to10 μg, corresponding to 5 × 10^4 ^to 10^5 ^GC) were subjected to 10% SDS-PAGE under reducing conditions, then the proteins were electrophoretically transferred from the gels onto Immobilon P membranes. These membranes were incubated for 1 h at room temperature with 20 mM Tris-buffered saline (TBS, pH 7.6), containing 3% BSA and 0.1% Tween-20 to saturate nonspecific sites. They were then incubated for 1 h at room temperature with anti-P-ERK1/2, or anti-ERK1/2, or anti-P450scc, or anti-3βHSD (final dilutions 1:1000) in TBS containing 1% BSA and 0.1% Tween-20. After washing in TBS containing 0.1% Tween-20, the membranes were incubated for 1 h at room temperature with horseradish peroxidase-conjugated anti-rabbit IgG (final dilution 1:10,000) in TBS containing 0.01% Tween-20, and the signal was visualized using ECL system followed by autoradiography. The autoradiograms were quantified using a videodensitometer (VDS-CL, Amersham Pharmacia Biotech).

### Actin staining and fluorescence microscopy

After culture in chamber slides, GC were fixed for 15 min with 4% paraformaldehyde in PBS. Cells were then washed with PBS and left in 0.1 M glycine in PBS for 15 min. After an additional wash, the cells were permabilized with 0.2% Triton X-100 (w/v) in PBS containing 1% BSA for 10 min, and nonspecific binding sites were blocked in 2% BSA. Cells were then treated for 30 min with 0.5 μM FITC-conjugated phalloidin. All the above incubations were performed at room temperature. After washing, cells were mounted in Mowiol and were studied under fluorescence microscopy. The percentage of the different morphological states of GC (spread cells, spindle-shaped cells, round cells) was established by counting 1000 to 1500 cells per culture well.

### Statistical analysis

All experimental data are presented as the mean ± SEM. Data were fitted to sigmoidal dose – response curves or Gaussian distributions with GraphPrad PRISM software (San Diego, CA, USA). The effects of increasing doses of inhibitors (cytochalasin D or PD98059) on cell numbers, percentages of round cells, thymidine labeling index and pyknotic index were analyzed using a one-way ANOVA for repeated measures followed by Tukey-Kramer tests. For a given substratum, the effects of the addition of anti-α6 IgG on cell numbers, percentages of round cells, thymidine labeling index and pyknotic index were analyzed using a paired *t *test. The effects of anti-α_6 _IgG or inhibitors (cytochalasin D or PD98059) on GC steroidogenesis were analyzed using a two-way ANOVA in order to assess the effects of treatment as well as of culture resulting from variations between both animals and the quality of the ovarian follicles dissected for each culture. Results from western immunoblotting analysis were analyzed by a paired *t *test. Comparisons with p > 0.05 were not considered significant.

## Results and discussion

### Consequences of α6β1 integrin activation by LN on GC shape and functions

**Figure 1:**
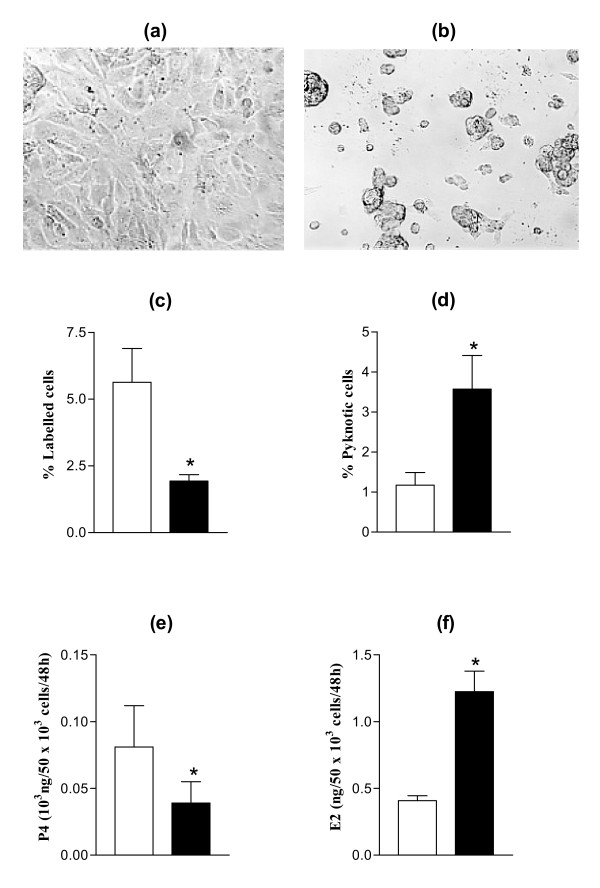
Effect of anti-α6 IgG on morphology and functions of GC cultured on LN substratum. GC were cultured up to 144 h on LN with (b and solid bars in c, d, e and f) or without (a and empty bars in c, d, e and f) anti-α6 IgG (0.5 μg/ml) in culture medium. (a) and (b): representative microscopical fields of cultured GC throughout the culture period; (c): proliferation rates of GC from small follicles, assessed by thymidine labelling at 48 h of culture; (d): pyknotic rates of GC from large follicles at 48 h of culture; (e): progesterone (P4) secretion by GC from large follicles between 96 h and 144 h of culture; (f): estradiol (E2) secretion by GC from large follicles between 96 h and 144 h of culture. Data represent mean ± SEM of 5 independent experiments. * : p < 0.01, with vs. without anti-α6 IgG.

Previous results have shown that LN, used as a culture substratum of ovine GC, induces cell spreading, enhances proliferation and survival rates, stimulates progesterone and reduces estradiol secretion by GC and that these effects are the consequence of α6β1 integrin activation by LN [6,14]. Accordingly, addition of anti-α_6 _IgG to the medium of GC cultured on LN induced dramatic cell rounding and important functional changes such as a decrease in proliferation rate and an increase in pyknotic rate of GC, as well as a decrease in progesterone and an increase in estradiol secretion (p < 0.01 for all parameters, Fig. 1). These effects were highly specific to the presence of LN in the culture substratum [6].

### Effects of cytoskeleton disruption by cytochalasin D on GC cultured on LN substratum

**Figure 2:**
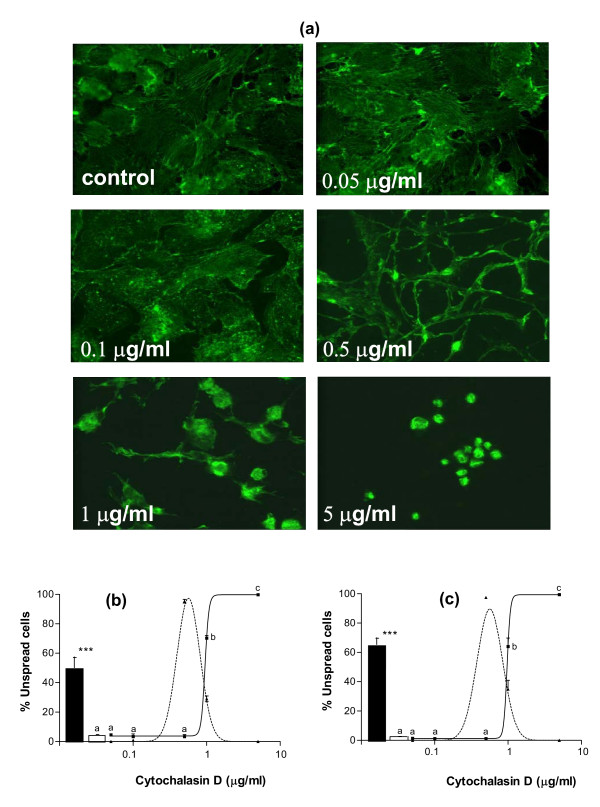
Effect of cytochalasin D on morphology of GC cultured on LN substratum. GC from small and large follicles were cultured for 48 h on LN with or without (control) cytochalasin D at different concentrations (between 0.05 and 5 μg/ml) in culture medium, and then actin was stained with FITC-conjugated phalloidin. (a): representative microscopical fields of cultured GC; GC spread on LN in control or in presence of low concentrations of cytochalasin D; most cells adopted a spindle-shaped morphology at 0.5 μg/ml of cychochalasin D, then rounded up at higher doses. (b) and (c): percentages of unspread cells, i.e. spindle-shaped (dashed line) and round (solid line) GC from small (b) and large (c) follicles; empty bars: percentages of round cells in control; solid bars: percentages of round cells with anti-α6 IgG (0.5 μg/ml) in culture medium. Data represent mean ± SEM of 3 independent experiments. In each graph, different letters indicate significant differences (p < 0.001); *** : p < 0.001, with vs. without anti-α6 IgG.

**Figure 3:**
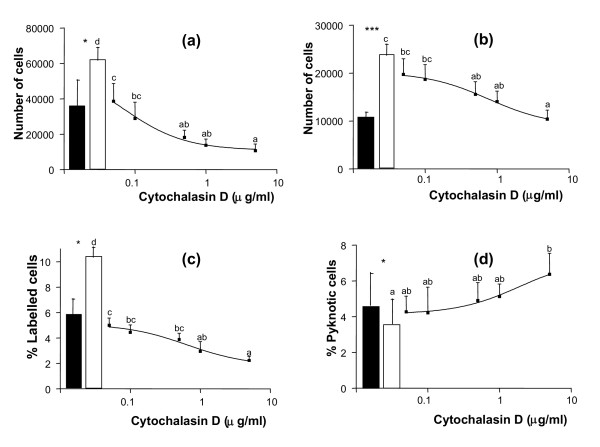
Effect of cytochalasin D on cell numbers, proliferation and pyknotic rates of GC cultured on LN substratum. GC from small (a, c) and large follicles (b, d) were cultured for 144 h on LN as described in legend of Figure 2. (a) and (b): numbers of cells at 144 h of culture; (c): proliferation rates of GC from small follicles at 48 h of culture; (d): pyknotic rates of GC from large follicles at 48 h of culture. Empty bars: control; solid bars: with anti-α6 IgG (0.5 μg/ml) in culture medium. Data represent mean ± SEM of 6 independent experiments. In each graph, different letters indicate significant differences (p < 0.05); * : p < 0.05, *** : p < 0.001, with vs. without anti-α6 IgG.

**Figure 4:**
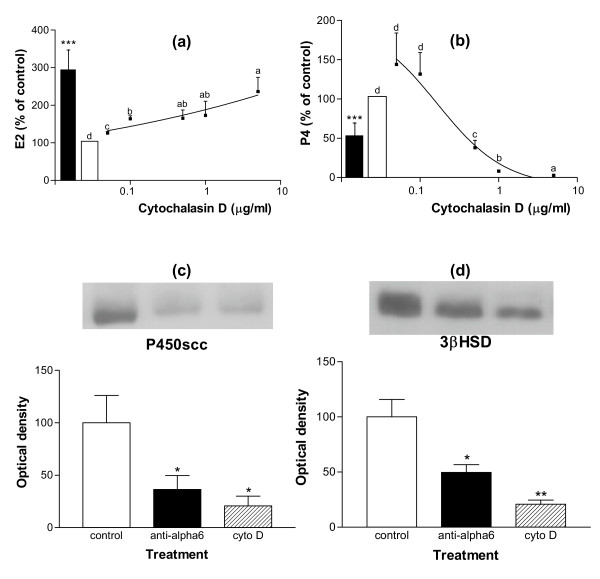
Effect of cytochalasin D on steroidogenesis of GC from large follicles cultured on LN substratum. GC from large follicles were cultured for 144 h on LN as described in legend of Figure 2. (a) and (b): estradiol (E2) and progesterone (P4) secretions between 96 h and 144 h of culture; data are expressed as percentages of control (100%, empty bars, corresponding to 0.40 ± 0.17 ng/50 × 10^5 ^cells/48 h for E2 and 700 ± 176 ng/50 × 10^5 ^cells/48 h for P4) and represent mean ± SEM of 5 independent experiments; solid bars: with anti-α6 IgG (0.5 μg/ml) in culture medium; in each graph, different letters indicate significant differences (p < 0.05); *** : p < 0.001, compared to control. (c) and (d): expression of P450scc and 3βHSD enzymes in GC at 144 h of culture, in control or in the presence of anti-α6 IgG or cytochalasin D (0.5 μg/ml) in culture medium; results show representative western immunoblotting experiments performed on 5 μg of GC extracts; data correspond to quantification of autoradiograms in arbitrary units (control mean = 100) and represent mean ± SEM of 5 independent experiments; * : p < 0.05, ** : p < 0.01, treated vs. control.

To assess whether all or part of these functional changes might be the consequence of cell rounding, the action of cytochalasin D, an inhibitor of actin polymerization, was studied on GC cultured on LN substratum. Addition of cytochalasin D to the culture medium of GC from small and large follicles reduced the formation of actin stress fibers and impeded cell spreading on LN, inducing the formation of spindle-shaped cells at doses higher than 0.1 μg/ml, and of round cells at doses higher than 0.5 μg/ml (p < 0.001 for GC from both small and large follicles, Fig. 2 a, b and 2 c). In GC from both small and large follicles, cytochalasin D treatment induced a clear dose-dependent decrease in cell numbers (p < 0.001 for GC from both small and large follicles, Fig. 3 a and 3 b). In cultures of GC from small follicles with a high proliferative activity, this decrease in cell number was associated with a dose-dependent decrease in cell proliferation rate (p < 0.001, Fig. 3 c). In cultures of GC from both small and large follicles, cytochalasin D also induced an increase in pyknotic rate at the 5 μg/ml dose (p < 0.05, Fig. 3 d). In cultures of GC from large follicles with a high steroidogenic activity, cytochalasin D induced a dose-dependent increase (p < 0.001) and decrease (p < 0.01) in secretion of estradiol and progesterone respectively (Fig. 4 a and 4 b). In accordance with the drop in progesterone secretion, a clear decrease in expression of the key steroidogenic enzymes P450scc (p < 0.05) and 3βHSD (p < 0.01) was also observed in cytochalasin-treated GC (Fig. 4 c and 4 d). All these effects of cytochalasin D on GC functions mimicked the actions of the anti-α_6 _IgG on GC cultured on LN substratum (Fig. 2, 3 and 4). Overall, cell rounding was associated with dramatic functional changes in GC. However, changes in proliferation rate and estradiol secretion were observed at cytochalasin D doses lower than 0.1 μg/ml, which did not induce observable changes in cell morphology.

From our results, cytochalasin D impeded ovine GC spreading on LN and induced cell rounding. These changes in cell shape were associated with a decrease in proliferation rate, an increase in cell death, a decrease in progesterone secretion and an increase in estradiol secretion. Previous investigations using inhibitors of actin microfilament polymerization, such as cytochalasin B or D, have shown the importance of actin cytoskeleton in GC functions. In GC, cytochalasin decreases actin and tropomyosin synthesis [29], causes the disorganization of microfilaments, intermediary filaments and microtubules [48] and induces cell rounding. These effects have been reported to mimick to some extent the effects of gonadotropins on the cytoskeleton [28,32,34]. To our knowledge, our study is the first report of the effects of cytochalasin on GC proliferation rate and cell death. In various cell models, such as kidney epithelial cells [49], hepatocytes [50] or carcinoma cells [51], cytochalasin has been shown to induce cell detachment from the substratum, to promote apoptosis and to inhibit cell proliferation. However, cytochalasin-induced changes in steroidogenesis are still a subject of controversy. Cytochalasin has been shown either to enhance (rat: [28,33,52]) or inhibit (hamster: [53], pig: [54], sheep: present study) progesterone secretion by GC and to inhibit it by luteal cells (cow: [55], pig: [56]). From the few data available concerning estradiol secretion, cytochalasin B is observed to block aromatase activity of hamsters GC [53] but to enhance it in immature rat Sertoli cells [57]. Whether these differences are the consequence of differences in cell types, animal species, or culture conditions is unknown. In any case, the functional effects of a disrupting drug such as cytochalasin must be considered with caution owing to its overall actions on cell metabolism and ion membrane exchanges. Moreover, experiments with cytochalasin have not allowed the specific role of LN-induced cytoskeleton changes on GC functions to be assessed. In our experiments, the observed effects of cytochalasin were likely to be the consequence of both cytoskeleton disruption and loss of interaction between cells and LN accompanying their detachment from the substratum.

### Effects of specific inactivation of the α6β1 integrin signalization pathway on GC cultured on LN and/ or RGD peptide substratum

**Figure 5:**
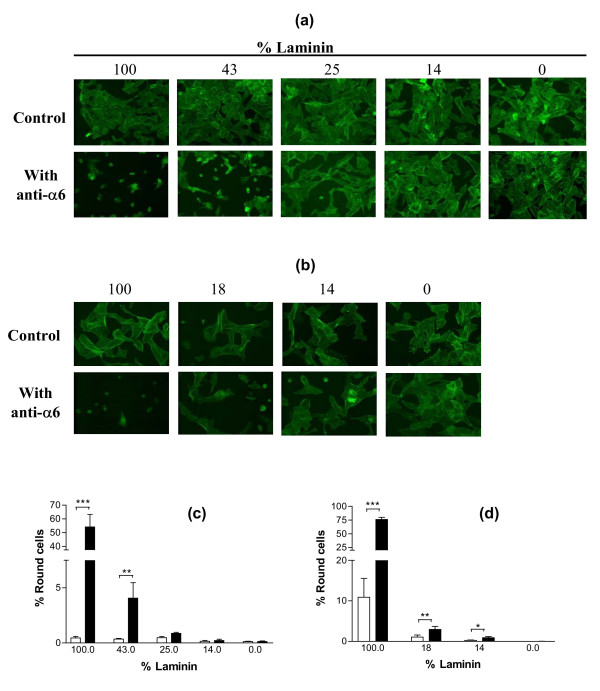
Effect of anti-α6 IgG on morphology of GC cultured on substrata containing different ratios of LN and RGD peptides. GC were cultured for 48 h on substrata containing percentages of LN varying between 100% (0% RGD peptides) and 0% (100% RGD peptides) in the mix, with or without anti-α6 IgG (0.5 μg/ml) in culture medium, and then actin was stained with FITC-conjugated phalloidin. (a) and (b): representative microscopic fields of cultured GC from small (a) and large (b) follicles. (c) and (d): percentages of round GC from small (c) and large (d) follicles; empty bars: control; solid bars: with anti-α6 IgG in culture medium; data represent mean ± SEM of 5 independent experiments; * : p < 0.05, ** : p < 0.01, *** : p < 0.001, with vs. without anti-α6 IgG.

**Figure 6:**
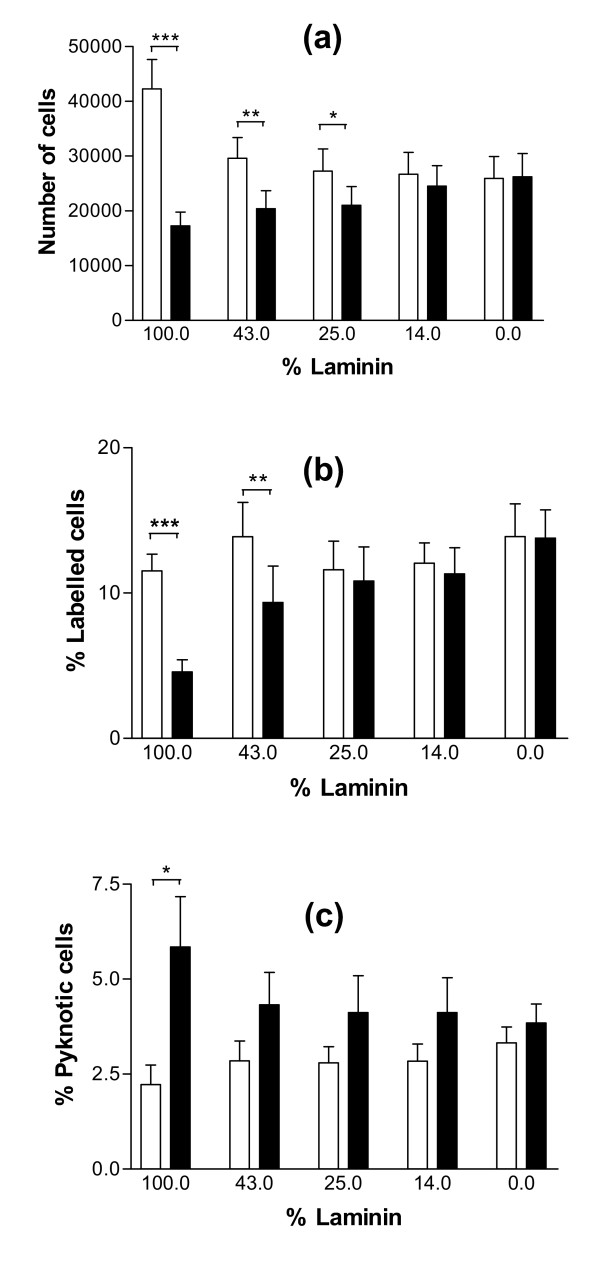
Effect of anti-α6 IgG on cell numbers, proliferation and pyknotic rates of GC from small follicles cultured on substrata containing different ratios of LN and RGD peptides. GC from small follicles were cultured for 144 h as described in legend of Figure 5. (a): numbers of cells at 144 h of culture; (b): proliferation rates of GC at 48 h of culture; (c): pyknotic rates of GC at 48 h of culture. Empty bars: control; solid bars: with anti-α6 IgG in culture medium. Data represent mean ± SEM of 6 independent experiments. * : p < 0.05, ** : p < 0.01, *** : p < 0.001, with vs. without anti-α6 IgG.

**Figure 7:**
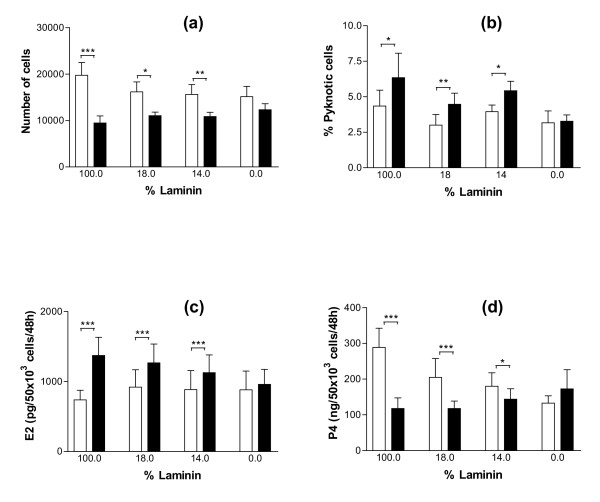
Effect of anti-α6 IgG on cell numbers, pyknotic rates and steroidogenesis of GC from large follicles cultured on substrata containing different ratios of LN and RGD peptides. GC from large follicles were cultured for 144 h as described in legend of Figure 5. (a): numbers of cells at 144 h of culture; (b): pyknotic rates of GC at 48 h of culture. (c) and (d): estradiol and progesterone secretions between 96 h and 144 h of culture. Empty bars: control; solid bars: with anti-α6 IgG in culture medium. Data represent mean ± SEM of 6 to 8 independent experiments. * : p < 0.05, ** : p < 0.01, *** : p < 0.001, with vs. without anti-α6 IgG.

**Figure 8:**
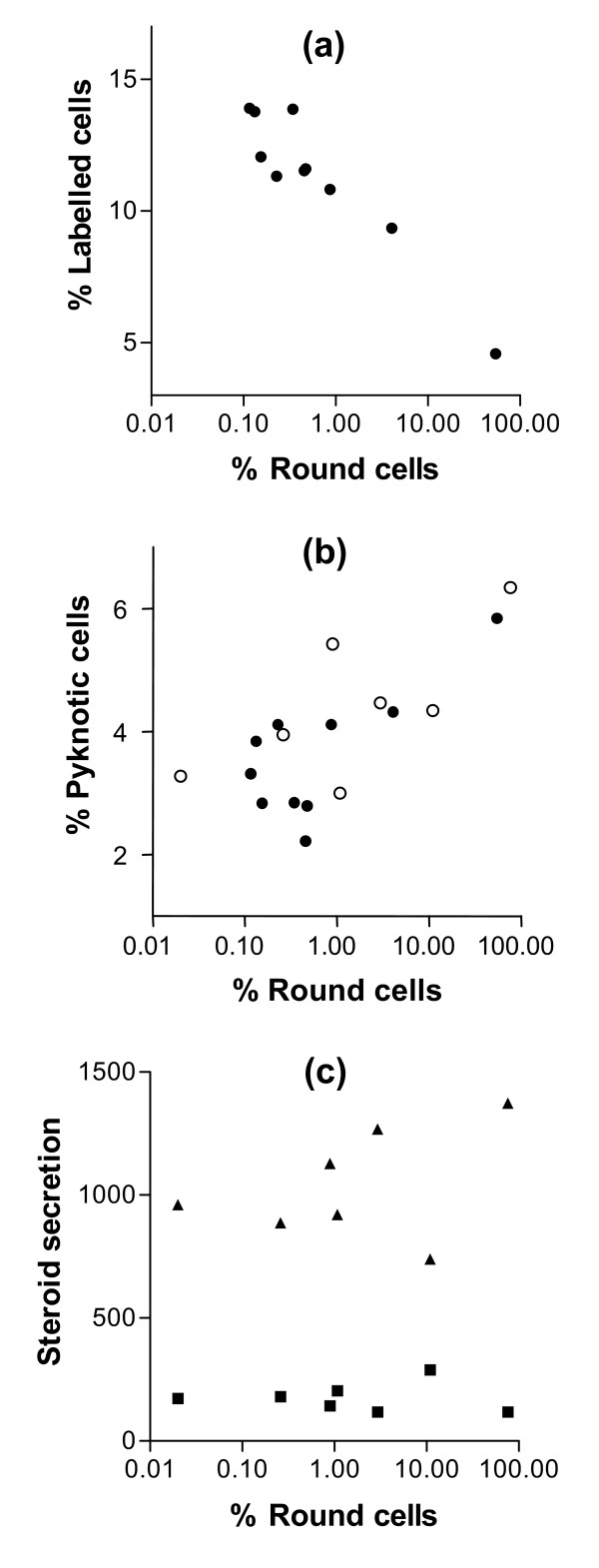
Relationships between percentages of round cells and proliferation rates, pyknotic rates or steroidogenesis of GC cultured on substrata containing different ratios of LN and RGD peptides. GC were cultured for 144 h as described in legend of Figure 5. (a): proliferation rates of GC from small follicles at 48 h of culture. (b): pyknotic rates of GC from small (solid circles) and large (empty circles) follicles at 48 h of culture. (c): steroid secretion by GC from large follicles between 96 h and 144 h; solid triangles: estradiol secretion, expressed in pg/50 × 10^3 ^cells/48 h, solid squares: progesterone secretion, expressed in ng/50 × 10^3 ^cells/48 h. Each data point is the mean of 7 independent experiments.

To further investigate the existence of a relationship between GC shape and function, an experiment was designed with the purpose of inactivating specifically the α6β1 integrin signalization pathway without changing GC shape. When cultured on substrata containing different ratios of LN and RGD peptides (varying between 100% and 0% LN), GC spread on all these different substrata (Fig. 5a and 5b). Addition of anti-α_6 _IgG in culture medium induced significant cell rounding only when the substratum contained at least 43% LN for cultures of GC from small follicles (p < 0.01), and 14% LN for cultures of GC from large follicles (p < 0.05) (Fig. 5 c and 5 d). In cultures of GC from small follicles, cell rounding induced by addition of anti-α_6 _IgG was associated with a decrease in cell number (p < 0.01 for cells cultured on a substratum containing at least 43% LN), a decrease in proliferation rate (p < 0.01 for cells cultured on a substratum containing at least 43% LN) and an increase in pyknotic rate (p < 0.05, only for cells cultured on 100% LN) (Fig. 6 a, b and 6 c). In cultures of GC from large follicles, cell rounding induced by addition of anti-α_6 _IgG was associated with a decrease in cell number (p < 0.01), an increase in pyknotic rate (p < 0.05), an increase in estradiol secretion (p < 0.001) and a decrease in progesterone secretion (p < 0.05), for cells cultured on a substratum containing at least 14% LN (Fig. 7 a, b, c and 7 d). Overall analysis of results of this experiment showed the existence of significant correlations between the percentage of round cells and the proliferation rate of GC from small follicles (r = -0.87, p < 0.001) on the one hand (Fig. 8a), and the pyknotic rate of GC from small follicles (r = 0.76, p < 0.05) and large follicles (r = 0.75, p < 0.05) on the other (Fig. 8b). In contrast, no significant correlation was found between the percentage of round cells and the amount of estradiol or progesterone secreted by GC from large follicles (Fig. 8c). These results support the existence of uncoupling of GC shape and steroidogenesis and suggest that mechanisms independent of cytoskeleton reorganization are also involved in α6β1 integrin-mediated LN actions on GC.

When GC spread on LN, cell proliferation and survival are clearly enhanced [13,14]. From our results, both proliferation and survival of GC cultured on LN, unlike steroidogenesis, were closely associated with cell shape and cytoskeleton organization. In other cell models, it has been established that cell spreading on ECM and associated changes in the actin cytoskeleton are necessary for progression through G1 and entry into the S phase of the cycle [50], and that signals elicited by the cytoskeleton act independently of ERK1/2 signals to drive the cell cycle machinery through the G1/S boundary and, hence to promote cell proliferation [58]. Interestingly, recent work has shown that the cytoskeleton orients the cell metabolic and signal transduction machinery, and that mechanical distortion of cells and the cytoskeleton through surface integrin receptors can switch cells between distinct gene programs (e.g. proliferation, differentiation and apoptosis) [59-61]. In ovarian follicles, GC lying directly on the basal lamina containing ECM adopt a columnar shape, whereas GC located in the follicular wall near the antral cavity are generally round [62-64]. These different cell morphologies are associated with different distributions of intracellular actin [65]. It can be hypothesized that these different cell shapes, resulting from cell interactions with ECM through integrin receptors, can directly influence GC functions. In particular, they could be responsible for establishing the gradient of survival and proliferation existing between the basal and the antral zone of the granulosa wall in ovarian follicles [64,66-68].

### Effects of specific inactivation of the ERK1/2 signalization pathway on GC cultured on LN

**Figure 9:**
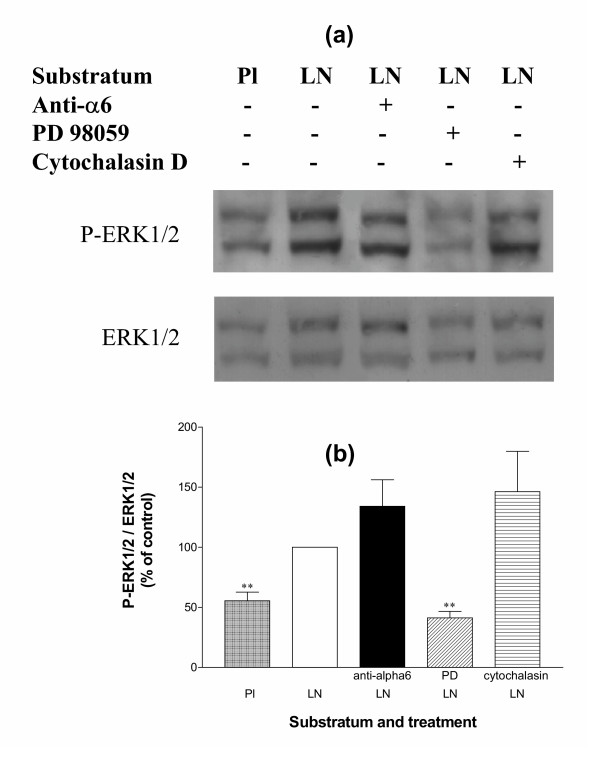
Expression and phosphorylation of ERK1/2 in GC cultured on LN or on plastic substratum. GC were cultured for 24 h on plastic (Pl) or on LN in the presence or absence of anti-α6 IgG (0.5 μg/ml), PD98059 (30 μM) or cytochalasin D (0.5 μg/ml) in culture medium. (a): representative western immunoblotting experiment performed on 10 μg of GC extracts. P-ERK1/2: phosphorylated ERK1/2. (b): ratio P-ERK1/2 / ERK1/2, obtained by quantification of autoradiograms in arbitrary units ; data are expressed as percentages of LN condition (100%, empty bar) and represent mean ± SEM of 6 independent experiments. **: p < 0.01, vs. LN.

To further study the mechanisms of action of LN on GC, we tested the importance of the ERK1/2 signalization pathway, which has been shown to transduce some of the α6β1 integrin-mediated effects of LN [36-39], in regulating both GC shape and functions. At 24 h of culture, phosphorylation of ERK1 and ERK2 was higher in cells cultured on LN than on the plastic substratum, indicating that LN might activate the ERK1/2 pathway in GC (p < 0.01, Fig. 9). Addition of PD98059, a specific inhibitor of the ERK1/2 activation pathway, inhibited ERK1/2 phosphorylation in GC cultured on LN substratum (p < 0.01) as expected, but the anti-α_6 _IgG and cytochalasin D had no effect (Fig. 9). The absence of effect of the function-blocking antibody raised against α6 subunit on ERK1/2 phosphorylation in GC cultured on LN was unexpected. It could be explained by the existence of other subunit integrins such as α3 and αv that are also able to bind LN [41] and have been shown to be present on GC ([19,69] and our unpublished observations). Whether these integrins participate in activation of the ERK1/2 signalization pathway when GC are attached to LN substratum remains to be studied. Alternatively, the ERK1/2 signalization pathway might respond poorly to α6β1 integrin activation, or the western immunoblotting method might not be sensitive enough to detect small quantitative changes in ERK1/2 phosphorylation.

**Figure 10:**
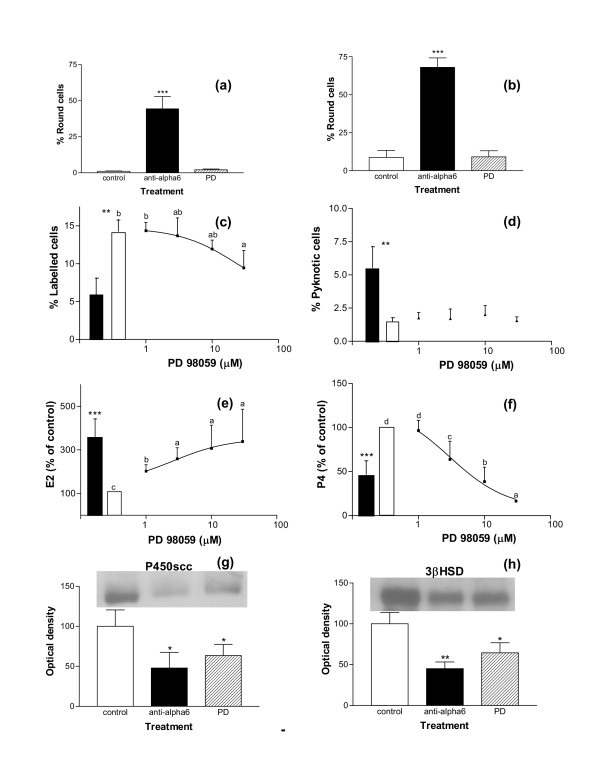
Effect of PD98059 on morphology, proliferation rates, pyknotic rates and steroidogenesis of GC cultured on LN. GC from small (a, c) and large (b, d, e, f, g, h) follicles were cultured for 144 h on LN in the presence or absence (control) of PD98059 at different concentrations (between 1 and 30 μM) in culture medium, and with or without anti-α6 IgG (0.5 μg/ml). (a, b): percentages of round cells at 48 h of culture; *** : p < 0.001, with anti-α6 IgG vs. control and PD98059-treated cells (30 μM). (c, d): proliferation rates and pyknotic rates of GC at 48 h of culture; empty bars: control; solid bars: with anti-α6 IgG in culture medium; in each graph, different letters indicate significant differences (p < 0.05); ** : p < 0.01, with vs. without anti-α6 IgG. (e, f): estradiol and progesterone secretions between 96 h and 144 h of culture; solid bars: with anti-α6 IgG in culture medium; data are expressed as percentages of control (100%, empty bars); in each graph, different letters indicate significant differences (p < 0.05) ; *** : p < 0.001 compared to control. (g, h): expression of P450scc (g) and 3βHSD (h) enzymes in GC at 144 h of culture, in control or in the presence of anti-α6 IgG or PD98059 (30 μM) in culture medium; results show representative western immunoblotting experiments performed on 5 μg of GC extracts; data correspond to quantification of autoradiograms in arbitrary units (control mean = 100); * : p < 0.05, ** : p < 0.01, treated vs. control. Data of the Figure represent mean ± SEM of 4 to 8 independent experiments.

In cultures of GC from both small and large follicles, PD98059 induced no significant change in GC spreading on LN (Fig. 10 a and 10 b) or in the pyknotic rate of GC, and had only a weak inhibiting effect on GC proliferation rate, acting at the highest dose of 30 μM (p < 0.05, Fig 10 c and 10 d). In contrast, PD98059 strongly increased estradiol secretion (p < 0.001) and decreased progesterone secretion (p < 0.001) as well as P450scc (p < 0.05) and 3βHSD (p < 0.05) expression in GC from large follicles cultured on LN (Fig. 10 e, f, g and 10 h). These effects were not observed when GC were cultured on plastic (data not shown). Overall, these results suggest that the ERK1/2 signalization pathway might be involved in LN actions on GC steroidogenesis, independently of LN actions on cell shape. In contrast, GC survival and proliferation are probably closely associated with cell shape and cytoskeleton organization.

In previous investigations, the ERK1/2 signalization pathway has been shown to be involved in the regulation of GC steroidogenesis, but its activation has either inhibiting or stimulating effects depending on the stimulus [70]. For example, ERK1/2 activation by gonadotropins inhibits Star protein expression and progesterone secretion, thereby contributing to desensitization mechanisms of hormonal action [71,72]. Likewise, prostaglandin F2alpha- or adenosine triphosphate-induced ERK1/2 activation inhibits hCG-stimulated progesterone secretion [73,74], whereas IGF-I- or melatonin-induced ERK1/2 activation increases it [75,76] and IGF-I-induced ERK1/2 activation enhances LDL receptor expression [77]. Interestingly, ERK1/2 have been shown to be intracellular signaling molecules that differentially regulate FSH-induced progesterone and estradiol synthesis in GC [78]. Thus, the divergent regulation of LN-induced progesterone and estradiol secretion by PD98059 observed in ovine GC supports the hypothesis that ERK1/2 is an important signalization pathway in the regulation of steroidogenesis by LN in GC.

## Conclusion

The results of this study emphasize the role of cytoskeleton and cell shape in controlling GC proliferation and survival. In GC, as in many other cell types, mechanotransduction processes strongly control these basic cell functions [60]. From our results, LN participates in this control by its α6β1 integrin-mediated actions on GC cytoskeleton. In contrast, steroidogenesis is a specific function of fully differentiated GC which is under the control of various signalization pathways of which the cytoskeleton would be of minor importance compared to the cAMP or ERK1/2 pathways. It is suggested that changes in cytoskeleton and cell shape induced by actions of gonadotropins, growth factors or ECM components would accompany rather than cause changes in steroidogenesis. Interestingly, the ERK1/2 pathway could play an important role in mediating the action of LN on GC luteinization.

## Authors' contributions

FLB, SF, CP and DM carried out the culture experiments together. FLB carried out the steroid assays and western immunoblotting experiments, participated in actin staining experiments, statistical analysis and interpretation of data, and participated in drafting the manuscript. SF participated in steroid assays and contributed to the conception and design of the study, the interpretation of data and the draft of the manuscript. CP carried out the thymidine labeling index and pyknotic index determination experiments and participated in actin staining experiments and interpretation of data. DM conceived the study, participated in its design and coordination, statistical analysis and interpretation of data and drafted the manuscript. FLB, SF and DM read and approved the final manuscript. CP died during preparation of the manuscript but her contribution was such that she deserves to be acknowledged as an author. This study is dedicated to her memory.
